# Molecular Mechanisms Driving Precision Medicine in Perioperative Care: Integrating Inflammation, Metabolism, and Neuroimmunomodulation for Personalized Outcomes

**DOI:** 10.3390/ijms262412043

**Published:** 2025-12-15

**Authors:** Ann-Kathrin Wenner, Lukas Andereggen, Markus M. Luedi

**Affiliations:** 1 Department of Anaesthesiology, Rescue- and Pain Medicine, Cantonal Hospital of St. Gallen, 9007 St. Gallen, Switzerland; ann-kathrin.wenner@h-och.ch; 2 Department of Neurosurgery, Kantonsspital Aarau, 5001 Aarau, Switzerland; lukas.andereggen@ksa.ch; 3 Faculty of Medicine, University of Bern, 3010 Bern, Switzerland; 4 Department of Anaesthesiology and Pain Medicine, Inselspital, Bern University Hospital, University of Bern, 3010 Bern, Switzerland

**Keywords:** translational medicine, personalized medicine, perioperative stress response, immunometabolism, surgical resilience, data-driven perioperative pathways

## Abstract

Precision perioperative medicine connects mechanisms across inflammation, metabolism, and neuroimmunomodulation to predict risk and individualize therapy. This review aims to incorporate landmark concepts and recent studies (2017–2025) as well as outlining how multi-omics and clinical analytics translate biology into actionable pathways. Key opportunities include cytokine-guided risk stratification, metabolic conditioning, and autonomic neuromodulation targeting the cholinergic anti-inflammatory reflex. Implementation requires robust phenotyping, interoperable data pipelines, and trials focused on functional recovery and cognition.

## 1. Introduction

Perioperative medicine faces a major challenge of attenuating the physiological stress response that occurs in every individual while simultaneously promoting an individualized recovery process. Globally, more than 300 million major surgeries are performed each year, and postoperative complications continue to represent a major, yet potentially preventable, source of morbidity and mortality [1,2]. Despite advances in anesthesia, hemodynamic optimization, and infection control, substantial inter-individual variability persists in outcomes such as organ dysfunction, infection, and neurocognitive decline [3,4,5]. These variations reflect complex interactions among immune activation, metabolic adaptation, and neural regulation—the three key systems coordinating the body’s response to surgical injury.

Out of an pathophysiological perspective, surgery triggers an integrated stress response encompassing inflammation, metabolic reprogramming, and neuroendocrine activation [6]. The emit of danger-associated molecular patterns (DAMPs) enhance pattern-recognition receptors such as Toll-like receptors and inflammasomes, initiating cytokine cascades, endothelial activation, and influence tissue perfusion [7,8,9]. Concurrently, metabolic shifts characterized by insulin resistance, enhanced lipolysis, and mitochondrial redox imbalance influence both immune competence and cellular repair [10,11]. Neural circuits, particularly the vagus-mediated cholinergic anti-inflammatory pathway, modulate these immune-metabolic processes and affect recovery trajectories, including postoperative delirium and cognitive dysfunction [12,13].

Epidemiologically, postoperative complications affect up to 20–30% of high-risk surgical patients and are responsible for nearly eight million deaths annually within 30 days of surgery [2,14]. Dysregulated inflammation, metabolic frailty, and neuroimmune dysfunction contribute substantially to this global burden, prolonging hospital stays and impairing long-term survival and quality of life [10,11]. Understanding the molecular mechanisms linking these interconnected systems is therefore essential for advancing perioperative precision medicine.

Current evidence demonstrates that standardized recovery protocols and evidence-based perioperative bundles improve mean outcomes but remain largely population-based [3,4]. Although biomarkers such as Interleukin-6, Interleukin-8, and C-reactive protein or indices of autonomic tone have been associated with adverse outcomes, clinical algorithms rarely integrate dynamic molecular or neuroimmune data [15,16,17]. Furthermore, omics-based profiling and neuroimaging studies have identified distinct biological endotypes—ranging from inflammation-heavy to metabolism-impaired phenotypes—suggesting that biologically personalized approaches are feasible [18,19,20].

However, the clinical implementation of mechanistic insights into actionable perioperative strategies remain incomplete. The integration of inflammatory, metabolic, and neuroimmunologic dimensions into precision frameworks is still in its early phase, limited by fragmented data systems, ethical constraints, and a lack of validated clinical tools [21].

This narrative review aims to synthesize the molecular mechanisms connecting inflammation, metabolism, and neuroimmunomodulation in the perioperative setting, to summarize emerging biomarkers and therapeutic targets, and to propose an integrative conceptual framework for personalized perioperative care.

Because this is a narrative review, literature was selected based on conceptual relevance and scientific contribution rather than through a predefined systematic protocol. We prioritized mechanistic and translational studies published between 2017–2025, identified through non-systematic searches in PubMed. In addition, we manually screened reference lists of key publications’ reference lists.

## 2. Inflammation: From Signals to Stratified Care

Surgical tissue damage triggers the release of endogenous danger signals (DAMPs), which are molecules that are emitted from dying cells [22]. These DAMPs activate pattern recognition receptors, including Toll-like receptors on immune and stromal cells [7,23]. This initiates intracellular signaling cascades including NF-κB, MAPK, and JAK/STAT, which drive the release of key cytokines (IL-1β, IL-6, IL-8, TNF-α) and modulate leukocyte migration and endothelial responses as well as vascular permeability to facilitate tissue repair [24,25]. However, when this system is dysregulated, this same response can become maladaptive, driving systemic inflammation and organ dysfunction—a central determinant of postoperative complications.

The inflammatory reflex further connects neural inputs to cytokine production and positions autonomic tone as a brake on excessive inflammation [26]. This feedback loop illustrates how inflammation does not act in isolation, but as part of an integrated system that also involves metabolic and neural pathways.

Beyond acute immune activation, interindividual differences in inflammatory magnitude are strongly influenced by age-related immune remodeling. Older adults often display a chronic, low-grade inflammatory state termed inflammaging, characterized by persistent activation of innate immune pathways, mitochondrial dysfunction, and the release of senescence-associated DAMPs [27,28]. This background inflammation amplifies perioperative cytokine cascades and delays immune resolution, predisposing older adults to pulmonary, cardiovascular, and cognitive complications through sustained immune dysregulation [29]. Recent work has emphasized that targeting immunosenescence and inflammaging may improve surgical resilience and reduce age-associated vulnerability to postoperative complications [30].

Defective production of specialized pro-resolving mediators (SMPs)—such as resolvins and protectins—further perpetuates inflammation, highlighting the therapeutic potential of resolution-promoting pharmacology in surgical patients [31]. Mechanistically, insufficient SPM generation leads to prolonged NF-κB activation and impaired termination of cytokine production; this disruption is typically reflected by sustained elevations in circulating IL-6 an IL-8. Moreover, immune-metabolic coupling plays a central role: hypoxia and nutrient shifts reprogram macrophage metabolism toward glycolysis via itaconate- and HIF-dependent mechanisms, sustaining cytokine output but depleting redox capacity [32]. Such metabolic rewiring produces a distinct downstream signature—characterized by IL-6-dominant cytokine release and itaconate-related metabolic intermediates—that is detectable through targeted metabolomics.

Understanding these intertwined pathways provides a biological basis for defining inflammatory endotypes. Here, we refer endotypes as reproducible patient subgroups that share a mechanistic alteration such as detective SPM biosynthesis or itaconate/HIF-driven metabolic reprogramming and an associated biomarker constellation encompassing cytokines (IL-6, IL-8), lipid-mediator patterns, and metabolic signatures. In practice, these endotypes can be identified by integrating cytokine trajectories with lipid mediator and metabolic profiles, enabling mechanistically informed stratification of perioperative immunomodulation.

Among circulating mediators, IL-6 and IL-8 are consistently associated with early complications after major surgery; in cardiac surgical cohorts, postoperative day-1 elevations predict pulmonary complications [15]. Beyond single markers, unbiased phenotyping reveals biological subgroups with distinct risks for postoperative pulmonary complications and resource use [33]. Composite indices merging inflammation and nutrition capture vulnerability and correlate with length of stay and morbidity after thoracic surgery [34].

These mechanistic and phenotypic insights directly inform strategies for perioperative immunomodulation. Perioperative immunomodulation ranges from multimodal analgesia and optimized hemodynamics to targeted anti-cytokine approaches [3,35]. Early concepts of multimodal pathways emphasized attenuation of the stress-inflammation cascade to accelerate recovery [36]. Neuromodulatory strategies (see below) offer complementary control over cytokine tone via the cholinergic anti-inflammatory reflex [26].

Collectively, these findings underscore the importance of integrating inflammatory profiling into perioperative risk assessment and monitoring. Measurement of IL-6, IL-8, and phenotype clusters should be used for risk stratification and adjustment of monitoring while postoperative dynamics are crucial for outcome assessment [15,33,34].

## 3. Metabolism: Energy Allocation and Hypoxic Adaptation

Surgical stress provokes and coordinates endocrine and metabolic response by elevating catecholamines, cortisol, and glucagon, driving lipolysis, proteolysis and gluconeogenesis [6]. Unregulated, this impairs the mechanisms of wound healing as well as immune competence. Therefore, early perioperative frameworks recognized that modulating this stress response improves recovery [36].

Beyond systematic catecholamine and cortisol surges, endocrine stress markers such as B-type natriuretic peptide (BNP) and NT-proBNP, the precursor protein, offer additional insight into cardiovascular adaption during surgical stress. Importantly, BNP is not only a marker of hemodynamic strain but also reflects impaired mitochondrial efficiency, reduced oxidative capacity, and altered substrate utilization under hypoxic or stress-related metabolic conditions. Experimental and clinical studies have shown that hypoxia an metabolic stress upregulate BNP expression via HIF-1α- dependent signaling, linking BNP release directly to cellular energy imbalance rather than purely to column or pressure overload [37].

Hahn et al. has shown that elevated perioperative BNP levels have been associated with increased morbidity and mortality, reflecting not only impaired hemodynamic resilience but also reduced metabolic stress tolerance and impaired mitochondrial-cardiac coupling. Genetic variants within the NPPA/NPPB loci modulate BNP expression and secretion, thereby linking genotype, ASA physical status, and cardiac vulnerability in the perioperative setting [38]. These findings underscore how endocrine and genomic determinants converge to influence individual stress responses—an essential concept in precision perioperative care.

In addition to cardiac and metabolic stress hormones, Copeptin, the C-terminal fragment of the arginine-vasopressin prohormone, provides an integrative measure of hypothalamic-pituitary activation. Elevated preoperative Copeptin concentrations have been associated with increased all-cause mortality after cardiac surgery, reflecting impaired neuroendocrine adaptation and systemic stress vulnerability [39]. Because vasopressin signaling closely interacts with glucose regulation, osmoregulation, and vascular tone, elevated Copeptin also reflects impaired metabolic stress handling and reduced capacity to main homeostasis under hypoxic and inflammatory conditions [40]. As a marker of vasopressin release, Copeptin reflects the interplay between stress signaling, hemodynamic stability and inflammatory regulation, strengthening its value as a biomarker for individualized perioperative risk assessment.

At the cellular level, Hypoxia-inducible factors (HIFs) act as central regulators of oxygen sensing and metabolic reprogramming and coordinates this with glycolysis, angiogenesis and mitophagy, regulating inflammatory set-points and cell survival under stress [20,41]. These pathways intersect immune function by shaping myeloid metabolism and barrier integrity.

Metabolic-immune crosstalk extends beyond classical energy substrates. The tryptophan-kynurenine pathway, meditated by Indoleamine 2,3-dioxygenase (IDO), represents a key immunometabolic regulator linking inflammation, redox balance, and neuroimmune communication. Elevated perioperative IDO activity has been associated with adverse outcomes following cardiac surgery, reflecting an immune-suppressive shift and impaired metabolic resilience [42]. This highlights IDO as a mechanistic biomarker and potential target for immunonutritional or pharmacologic modulation within precision perioperative strategies.

In addition to amino-acid and redox metabolism, alterations in lipid homeostasis reflect another layer of perioperative metabolic adaptation. Decreases in circulating cholesterol and triglycerides after major surgery have been linked to inflammatory activation and delayed recovery, suggesting that lipid availability modulates both energy and immune response [43].

Recent studies have emphasized that mitochondrial efficiency and bioenergetic flexibility are central determinants of perioperative resilience, with oxidative stress and reperfusion injury representing key mechanisms of postoperative organ dysfunction [44,45]. Mitochondrial dysfunction, precipitated by surgical hypoxia and oxidative stress, disrupts ATP synthesis and shifts metabolism toward anaerobic glycolysis, thereby exacerbating systemic inflammation and perioperative organ injury [46]. Interventions that preserve mitochondrial function—such as exercise-based prehabilitation, targeted antioxidant strategies, and modulation of mitophagy through PGC-1α signaling—have shown promise in improving postoperative recovery and functional capacity [47]. Recent translational work further supports that optimizing mitochondrial bioenergetics through structured rehabilitation and metabolic conditioning enhances oxidative capacity and may accelerate postoperative recovery [48]. Moreover, mitochondrial–immune crosstalk is increasingly recognized as bidirectional: inflammatory cytokines impair mitochondrial biogenesis, while mitochondrial danger signals (mtDAMPs) further propagate immune activation [49]. This interplay provides a mechanistic link between metabolic control, immune tone, and postoperative recovery, underscoring mitochondria as both biomarkers and therapeutic targets within precision perioperative medicine.

Metabolomic analyses demonstrate substantial heterogeneity in redox balance, fatty acid oxidation, and amino acid metabolism, especially in individuals with frailty and sarcopenia [10,11]. HIF signaling pathways have been implicated in enhanced resilience during surgical prehabilitation, suggesting a mechanistic link between metabolic fitness and immunological recovery [41,50].

Building on these insights, metabolic prehabilitation, which combines nutritional optimization, exercise conditioning, and psychological support before surgery, has shown promise in enhancing physiological reserve and reducing postoperative complications [51].

These interventions aim to prime mitochondrial function, improve substrate flexibility, and attenuate inflammatory overdrive. Personalized nutrition, including adequate protein intake, omega-3 supplementation, carbohydrate loading and minimized fasting complement pharmacologic approaches that modulate HIF/mTOR axes. Inflammation–nutrition indices may guide escalation or de-escalation of these strategies [34].

Together, these concepts underline the central role of metabolic adaption in perioperative resilience.

## 4. Neuroimmunomodulation: CNS—Immune Crosstalk and Therapeutics

The autonomic nervous system influences immune regulation, integrating neural and inflammatory responses through bidirectional signaling. One key component is the vagus nerve, that plays a central role in modulating cytokine production.

The vagus nerve regulates cytokine production through the cholinergic anti-inflammatory reflex, where acetylcholine binding to α7-nicotinic receptors on macrophages reduces the production of TNF-α and IL-6 [26]. Heart rate variability (HRV) represents a non-invasive marker of autonomic balance and has been linked to both cardiovascular vulnerability and inflammatory status—a marker and a potential therapeutic target [52].

The disturbance of these regulatory networks promotes postoperative neuroinflammation and cognitive decline. Blood–brain barrier perturbations, microglial activation, and disrupted synaptic signaling contribute to postoperative delirium and neurocognitive disorders [53]. Analgesic strategies that better align with nociceptive physiology have reduced delirium in elderly patients undergoing major orthopedic surgery [54].

Recent advances in neuromodulation have opened new avenues for therapeutic intervention. Non-invasive techniques such as transcutaneous vagus nerve stimulation (tVNS) and digital neuromodulators are under investigation for perioperative use, leveraging the cholinergic anti-inflammatory reflex [12,13,26]. Activation of α7-nAChR receptors on macrophages suppresses TNF-α and IL-6, potentially reducing postoperative delirium and improving recovery [12,13]. Mendelian randomization has revealed a causal link between insulin therapy, delirium, and cognitive decline, highlighting neuroimmune–metabolic interactions as a therapeutic target [55]. Functional neuroimaging approaches have identified brainstem regions that coordinate vagal activity, offering a neuroanatomical basis for targeted neuromodulation in perioperative settings [16]. Moreover, autonomic balance and HRV are associated with resilience to surgical stress and may serve as both predictive biomarkers and therapeutic targets [52].

Emerging evidence also underscores the role of hypothalamic–pituitary–adrenal (HPA) axis activity and circadian synchronization in shaping perioperative neuroimmune outcomes [56]. Dysregulated cortisol rhythms and blunted diurnal variability have been linked to exaggerated inflammatory responses and postoperative delirium, particularly in older and critically ill patients [57]. The HPA axis interacts closely with autonomic circuits, coordinating glucocorticoid feedback with vagal tone to fine-tune immune responses under stress [58]. Circadian misalignment—resulting from disrupted light exposure, anesthetic timing, or sleep deprivation—alters leukocyte trafficking and cytokine expression, thereby impairing immune resolution and wound healing [59]. Restoring physiological rhythmicity through optimized perioperative light–dark cycles, timing of analgesic administration, and maintenance of sleep–wake architecture has shown potential to mitigate neuroinflammation, support cardiovascular stability, and enhance recovery after major surgery [60]. These findings extend the scope of perioperative precision medicine by integrating neuroendocrine timing and autonomic balance as actionable determinants of individualized outcomes.

To validate these mechanisms clinically, integrated monitoring frameworks integrating HRV analysis, inflammatory biomarker panels, and symptom trajectories can verify neuromodulation target engagement and guide individualized dose titration of neuromodulatory therapy [15,52]. Together, these advances mark the transition from descriptive understanding to actionable strategies of improving perioperative outcomes.

## 5. Systems Integration: Omics, AI, and Clinical Pathways

The establishment of multi-omics, artificial intelligence, and data-driven clinical pathways represent a transformative step toward precision perioperative medicine.

Integration of genomic, transcriptomic, proteomic, metabolomic and epigenomic data enables the identification of biological endotypes. Genetic variants influence stress responses, pharmacogenomic profiles shape drug sensitivity and transcriptional shifts reveal immune and metabolic adaption during surgical stress [18,61]. Proteomic snapshots of cytokines, complement factors, and coagulation cascades identify patients prone to systemic inflammation, while metabolomic signals such as altered amino acid handling or ceramide buildup forecast poor recovery or organ dysfunction [11,62].

When multiple molecular layers are analyzed together, patients can be grouped into these biological endotypes that distinguish in prognosis and therapeutic needs [63]. Research in sepsis has demonstrated that molecular signatures can inform prognosis and guide therapeutic decisions—an approach translatable to perioperative care [19,20]. Similar strategies could classify perioperative patients into inflammation-heavy, metabolism-impaired, or neuroimmunologically vulnerable subgroups—providing the rationale for tailored therapies such as immunonutrition, HIF-modifying substances, or neuromodulation.

To address how multi-omics and AI are integrated (rather than treated as parallel tracks), we now explicitly describe their combined use. Recent work shows that multi-omics datasets (e.g., transcriptomic, proteomic, metabolic modules) can be fused using advanced machine-learning architectures—including early-fusion neural networks, stacked ensemble models, and deep multimodal frameworks—to derive actionable perioperative endotypes and robust prediction scores. For example, integrative models combining diverse omics layers have, in multiple contexts, outperformed single-layer data analyses and enabled mechanistically grounded risk prediction (e.g., inflammation- or hypoxia-dominant trajectories). These findings provide a practical template for how multi-omics profiling and AI-driven analytics can converge in perioperative medicine [64].

Predictive models combining continuous physiological data with molecular markers may detect impending complications hours ahead of clinical manifestation.

As an example, machine-learning models trained on hemodynamic patterns and supplemented with preoperative risk profiles could enable earlier identification of sepsis or delirium compared to conventional monitoring [65,66].

For meaningful clinical adoption, decision-support systems should seamlessly integrate into established perioperative workflows while delivering precise and actionable recommendations that can directly inform patient management—for example, ‘initiate tVNS in an HRV-low, IL-6-high profile’—and being adapted dynamically as new data become available [67,68].

Within the framework, systems biology and network medicine provide conceptual depth. Systems maps of immune, metabolic, and neural interactions can reveal control points with disproportionate clinical relevance. Analyses repeatedly place IL-6 and HIF-1α near the center of immune-hypoxia crosstalk, highlighting them as pivotal regulators [50,69].

Overlaying omics-based networks with outcome data shifts research from description to mechanistic hypothesis generation.

The translation of multi-omics and AI-based insights into perioperative decision-making requires robust validation pipelines that bridge discovery science and bedside application [18]. Prospective studies integrating molecular stratification with adaptive clinical trial designs have demonstrated how biologically defined patient subsets respond differently to standardized interventions, providing early proof-of-concept for precision-guided perioperative management [70]. Collaborative data ecosystems, such as federated learning frameworks, allow models to be trained on distributed hospital datasets without compromising patient privacy—an essential prerequisite for generalizable, bias-resistant algorithms [71]. Moreover, integrating continuous physiological monitoring with real-time molecular readouts through wearable biosensors enables dynamic feedback control of inflammatory and metabolic states [72]. Such approaches signal the emergence of a learning perioperative system that adapts therapeutics to individual biological trajectories rather than fixed clinical protocols.

Despite these advances, the integration of omics into real-time perioperative care remains highly challenging. Within clinical practice, data management represents a major obstacle. Genomic and neurocognitive data are particularly sensitive, requiring early consideration of consent, privacy, and equitable access. Algorithms must be validated across diverse patient populations to prevent amplification of bias [73]. Sustainable implementation further depends on close interdisciplinary collaboration, bringing together anesthesiologists, surgeons, intensivists, data scientists, and ethicists. Embedding pilot projects within existing clinical routines—so-called “Learning Health Systems”—allows for iterative refinement of care pathways while simultaneously generating generalizable evidence (Table 1) [74].

## 6. Clinical Translation and Trials

To explicitly operationalize the systems-integration framework outlined in Part 4, clinical translation requires that each perioperative strategy be anchored in a measurable biological endotype and supported by real-time analytics. For example, inflammation-dominant endotypes defined by IL-6/IL-8 trajectories and lipid mediator profiles can trigger cytokine-guided multimodal analgesia or targeted anti-inflammatory adjuncts; hypoxia- or metabolism-impaired endotypes identified through HIF-1α signatures, ceramide patterns, or metabolomic fingerprints can guide individualized metabolic prehabilitation or mitochondrial-protective strategies; and neuroimmune-vulnerable endotypes defined by low HRV, dysregulated cortisol rhythms, or α7-nAChR expression may inform neuromodulation or chronotherapy protocols. These endotype-driven interventions are increasingly supported by rapid molecular diagnostics, multimodal data fusion, and adaptive AI-based decision-support models, which together enable the conceptual integration described above to be directly applied at the bedside.

The translation of mechanistic insights into perioperative precision medicine requires carefully designed clinical studies that can accommodate biological heterogeneity. Traditional randomized controlled trials, while foundational, often dilute therapeutic signals because they group together patients with fundamentally different biological risk profiles. Adaptive trial designs offer an important solution to this challenge. Platform studies, for example, allow multiple interventions to be tested in parallel within a single infrastructure, while basket designs stratify patients not by the type of surgery but by biological signatures such as elevated IL-6 or reduced HRV. Response-adaptive randomization further increases efficiency by reallocating participants toward more promising interventions as early data emerge, reducing both time and patient exposure to less effective strategies. These innovative designs mirror developments in oncology and hold promise for perioperative medicine by aligning treatments with specific biological endotypes [20,33].

The first examples of precision perioperative pathways are beginning to take shape. Inflammation-guided analgesia, triggered specifically in patients with an inflammation-dominant endotype (elevated IL-6/IL-8 clusters or SPM-deficient signatures), illustrates how omics-defined biology and continuous autonomic analytics can be combined to tailor multimodal pain management while minimizing opioid exposure [13,52]. Nutritional and exercise-based prehabilitation becomes particularly relevant in patients with a hypoxia- or metabolism-impaired endotype, where ceramide patterns, HIF-1α activity, or redox-stress signatures indicate impaired metabolic resilience [34]. Metabolic prehabilitation, which combines nutritional optimization, exercise conditioning, and psychological support before surgery, has shown promise in enhancing physiological reserve and reducing postoperative complications [51]. Neuromodulatory approaches such as transcutaneous vagus nerve stimulation or pharmacologic α7-nAChR activation are being tested in patients with a neuroimmune-vulnerable endotype—characterized by low HRV, dysregulated cortisol rhythms, or impaired α7-nAChR signaling—to attenuate perioperative cytokine surges and support cognitive recovery [12,13,16]. Drug repurposing has also shown value, as illustrated by selective COX-2 inhibition with Parecoxib, which not only provides analgesia but also modulates neuroinflammatory circuits to reduce postoperative delirium [75]. These examples demonstrate how mechanistic understanding can translate into targeted perioperative interventions.

To clarify how multi-omics an AI-based tools integrate across these clinical components, we now explicitly describe their role in linking biological heterogeneity to individualized perioperative strategies. Multi-omics profiles can define mechanistic endotypes (e.g., inflammation-dominant, hypoxia-dominant, or metabolic-impaired), which can identify patient subgroups most likely to benefit from specific interventions. AI-based decision-support systems can further incorporate these endotypes together with real-time physiologic data (e.g., HRV, cytokine trajectories) to guide the timing, selection, or intensity of interventions—such as immunonutrition, vagal neuromodulation, targeted anti-cytokine therapy, or metabolic conditioning—with dynamic adjustment as new data emerge. This integration clarifies how omics-derived signatures and machine-learning models act as the connective tissue between biological discovery and individualized clinical management.

However, the successful implementation of precision approaches in everyday clinical practice requires more than novel trial designs or therapeutic concepts. Diagnostics must deliver actionable information within hours, not days. Rapid cytokine assays and bedside HRV monitoring are crucial to enable timely decisions. Decision-support tools should be embedded directly into anesthesia monitors and electronic health records, presenting concise, interpretable recommendations rather than raw data streams. Equally important is clinician training: anesthesiologists, surgeons, and intensivists must learn to interpret omics-based and computational outputs in a clinically meaningful way. Finally, scalability and equity are essential considerations. Precision pathways must be adaptable to a variety of resource settings to avoid widening disparities in surgical care.

Recent advances in systems biology and data integration provide a foundation for real-time precision medicine. Multi-omics profiling combined with digital biomarkers such as HRV and inflammatory signatures enables continuous characterization of patient trajectories rather than static risk assessment [18]. Integrating these biological data streams into machine-learning frameworks supports adaptive therapeutic feedback, where interventions such as targeted immunonutrition, neuromodulation, or metabolic prehabilitation can be titrated according to evolving physiological states [76]. Early clinical pilots demonstrate the feasibility of coupling omics-derived insights with bedside monitoring to dynamically guide analgesia, fluid management, and immune modulation [72]. Such adaptive systems mark a transition from guideline-based to biologically responsive perioperative care, aligning mechanistic discovery with clinical implementation.

To illustrate practical implementation, a precision perioperative pathway can follow a reproducible, four-step workflow: (1) Rapid phenotyping: bedside cytokine panels (IL-6/IL-8), targeted metabolomics (lactate, amino acids, ceramides), and continuous HRV/autonomic monitoring generate an initial biological endotype within hours. (2) Multimodal data fusion: omics, physiological, and clinical variables are combined through early-fusion neural networks or stacked ensemble models to refine risk prediction. (3) Endotype-triggered interventions: predefined decision rules (e.g., “inflammation-dominant → cytokine-guided analgesia,” “hypoxia-dominant → metabolic prehabilitation or HIF/mTOR-modulating strategies,” “neuroimmune-vulnerable → tVNS, circadian optimization”) initiate targeted therapy. (4) Adaptive feedback: real-time HRV, cytokine trajectories, and digital biomarkers continuously update the patient’s trajectory, enabling dynamic titration of interventions. This workflow exemplifies how omics-derived endotypes and AI-based analytics can be integrated into routine perioperative care.

Looking forward, perioperative precision medicine will benefit from hybrid trial designs that combine pragmatic and adaptive elements, striking a balance between generalizability and biological specificity. Wearable devices capable of continuous autonomic and metabolic monitoring before and after surgery will likely enrich risk assessment and recovery tracking. Longitudinal studies are needed to connect perioperative biological signatures not only to immediate postoperative morbidity but also to long-term outcomes such as cognitive decline, progression of frailty, and cardiovascular events. Multicenter consortia and standardized biomarker panels will be essential for ensuring reproducibility and facilitating meta-analysis.

Clinical translation is the critical bridge between mechanistic discovery and individualized patient care. Through adaptive trials, targeted interventions, and thoughtful implementation, precision perioperative medicine can move from theoretical promise to routine practice, ultimately improving recovery trajectories for diverse patient populations.

Future integration of omics-derived, physiological, and digital biomarkers will enable perioperative care to evolve from standardized management toward dynamic, biology-guided precision pathways.

## 7. Conclusions

In sum, inflammation, metabolism, and neuroimmunomodulation shape perioperative trajectories. Advances in cytokine phenotyping, metabolic profiling, and autonomic monitoring provide tools to identify patient subgroups at highest risk for adverse outcomes. Integration of these biological signals through multi-omics platforms and network-based approaches enables the definition of mechanistic endotypes that may guide therapy selection. Machine learning and explainable AI offer the potential to merge complex datasets with clinical variables, generating actionable predictions at the bedside.

Yet, translation into practice requires more than scientific discovery. Adaptive trial designs—including platform and basket approaches—are necessary to test tailored interventions efficiently, while pragmatic elements ensure generalizability. Early examples already demonstrate the promise of inflammation sensitive analgesia, metabolic prehabilitation, neuromodulatory strategies, and repurposed drugs. Future work must include patient centered outcomes such as long-term recovery, including cognition and functional independence, as well as short-term morbidity.

For implementation, infrastructure and administration are key: rapid diagnostics, interoperable data systems, and ethical oversight must accompany science. Equally important is the establishment of learning health systems in which anesthesiologists, surgeons, intensivists, data scientists, and ethicists collaborate seamlessly.

Taken together, precision perioperative medicine requires a shift from one-size-fits-all protocols to biologically informed, dynamically adaptive care. By aligning mechanistic insights with real-world practice, the field can move toward truly personalized outcomes—where surgical recovery is optimized not for the “average” patient, but for each individual.

## Figures and Tables

**Table 1:** Biological Systems in Perioperative Precision Medicine: Summary of the central biological systems underlying precision perioperative medicine, highlighting molecular pathways, measurable biomarkers, and corresponding targeted strategies.

System	Key Mechanisms	Representative Biomarkers	Therapeutic/Monitoring Approaches	Recent Advances
Inflammation	DAMP/TLR signaling, NF-κB, JAK/STAT activation, cytokine cascade	IL-6, IL-8, TNF-α	Cytokine-guided analgesia, perioperative immunomodulation, vagus-nerve–based control	Integration of inflammatory indices with AI-based risk models; rapid bedside cytokine assays
Metabolism	HIF-1α signaling, mitochondrial bioenergetics, endocrine stress response	Lactate, amino acids, ceramides, cortisol	Nutritional optimization, metabolic prehabilitation, HIF/mTOR modulation	Multi-omics assessment of metabolic resilience; metabolomic fingerprints guiding prehabilitation
Neuroimmunomodulation	Cholinergic anti-inflammatory reflex, HPA axis, circadian regulation	HRV, cortisol rhythm, α7-nAChR expression	Transcutaneous VNS, chronotherapy, autonomic modulation	Coupling HRV and inflammatory panels for real-time feedback; circadian optimization of analgesia
Systems Integration	Multi-omics, AI-assisted phenotyping, network medicine	Composite biological endotypes	Machine-learning–based decision support, adaptive trial design	Federated learning frameworks; wearable biosensors enabling continuous monitoring

## Data Availability

No new data were created or analyzed in this study. Data sharing is not applicable to this article.

## Abbreviations

The following abbreviations are used in this manuscript:

AI	Artificial Intelligence
ASA	American Society of Anesthesiologists
BNP	B-type Natriuretic Peptide
CNS	Central Nervous System
COX-2	Cyclooxygenase-2
DAMPs	Damage-Associated Molecular Patterns
HIF	Hypoxia-Inducible Factor
HIF-1α	Hypoxia-Inducible Factor 1 Alpha
HRV	Heart Rate Variability
IDO	Indoleamine 2,3-Dioxygenase
IL	Interleukin
IL-1β	Interleukin-1 Beta
IL-6	Interleukin-6
IL-8	Interleukin-8
JAK/STAT	Janus Kinase/Signal Transducer and Activator of Transcription
MAPK	Mitogen-Activated Protein Kinase
mTOR	Mechanistic Target of Rapamycin
NF-κB	Nuclear Factor Kappa-Light-Chain-Enhancer of Activated B Cells
NPPA/NPPB	Natriuretic Peptide A/Natriuretic Peptide B Genes
NT-proBNP	N-terminal pro–B-type Natriuretic Peptide
tVNS	Transcutaneous Vagus Nerve Stimulation
TNF-α	Tumor Necrosis Factor Alpha
